# Optimal Flow—A Pilot Study Balancing Sheep Movement and Welfare in Abattoirs

**DOI:** 10.3390/ani11020344

**Published:** 2021-01-29

**Authors:** Melissa J. Starling, Elyssa Payne, Paul McGreevy

**Affiliations:** Sydney School of Veterinary Science, University of Sydney, Camperdown, NSW 2050, Australia; elyssa.payne@gmail.com (E.P.); paul.mcgreevy@sydney.edu.au (P.M.)

**Keywords:** herding dogs, working dogs, livestock handling, livestock stress, animal welfare, lairage, abattoir

## Abstract

**Simple Summary:**

Sheep in Australia are transported to abattoirs for slaughter by trucks and usually left in lairage (holding pens) overnight. They are then moved through the abattoir via a series of pens that ultimately leads to a single file race immediately before slaughter. This movement has the potential to induce considerable stress among the sheep, thus compromising welfare. This study introduces the concept of ‘Optimal Flow’, where sheep movement through the abattoir is the most efficient balance between speed and minimising overt signs of distress in sheep. The results of the pilot study suggest that Optimal Flow at this abattoir occurs when sheep are given enough space to move freely.

**Abstract:**

Abattoirs are faced with the challenge of moving livestock efficiently through the plant, while also engaging in handling practices that assure good animal welfare. Achieving optimal outcomes for both of these goals can bring them into conflict. An additional source of conflict can arise from the design of the abattoir. These problems are compounded by the dearth of research available to inform how livestock should be handled to achieve all of these goals. We applied the concept of ‘Optimal Flow’ to describe conditions under which rate of movement is maximised while overt signs of distress in sheep are minimised. Effectively, this represents the point at which trade-offs between speed and welfare converge. The current pilot study examined the behavioural interactions between humans (*n* = 5), livestock herding dogs (*n* = 7), and sheep (*n* = 3235) in a large Australian abattoir to describe the factors associated with an increase or decrease in rate of sheep movement per minute. It revealed that distress behaviours in sheep were associated with dog presence and with a decrease in livestock movement rate. However, we found that as sheep density increased, there was increased livestock movement rate as well as an elevated incidence of distress behaviours. Optimal Flow at this abattoir was achieved by maintaining sheep at lower densities. Our report discusses the possible confounds in this interpretation.

## 1. Introduction

Sheep in Australia typically travel to abattoirs on trucks, and the associated movement, noise, and ambient temperature flux can make travel stressful for them [1] and thus compromise carcass quality [2]. The impact of these stressors may be mitigated by moving sheep from trucks into lairage where they are left to rest and recover overnight before slaughter [3]. The following day, sheep are moved from lairage to the adjacent abattoir for processing. Abattoirs in Australia do not follow a standard design, but typically there is a forcing pen adjacent to a single-file race. The forcing pen is designed to funnel the sheep towards a single-file race so that they enter it one at a time, prior to entering the so-called knocking box (where slaughter occurs).

It is important for the quality of the meat and for animal welfare goals that any distress the sheep are exposed to is kept to a minimum while they are moved through the processing plant [4]. At the same time, the speed with which sheep can be moved from lairage to the single-file race has considerable impact on the rate at which the rest of the abattoir can process carcasses, so providing a steady flow of animals is another reason animals remain in lairage [3]. Therefore, we were interested in the combination of steady movement and minimal distress to sheep that represents ‘Optimal Flow’—the intersection of productivity goals and welfare goals.

Sheep may be reluctant to move forward in the unfamiliar surroundings of an abattoir [5]. This can be overcome, depending on the layout of the plant, by pressuring sheep to move forward by presenting aversive stimuli that they are motivated to move away from. Such stimuli vary from plant to plant, but they include humans with noise-maker devices, such as plastic bags or bells, which emit auditory stimulus that sheep tend to move away from, or by using livestock herding dogs specially bred and trained to control sheep in small spaces. Dogs are predatory animals, and sheep instinctively move away from them [6], but working dogs may also bark and harass sheep at close quarters to make sheep move away. Our own informal surveys have shown that abattoirs in Australia may employ 2–10 dogs per shift, depending on the abattoir design and the volume of sheep processed each day (unpublished data). Livestock herding dogs can be used in abattoirs to move sheep from one pen to another, to pressure them past obstacles in the abattoir design that cause sheep to hesitate or balk, to break-up groups of sheep that have bunched very tightly into a corner, and to maintain steady pressure to facilitate movement through bottlenecks in the abattoir. In Australia, dogs are not commonly used in abattoirs to herd livestock species other than sheep.

**Figure 1:**
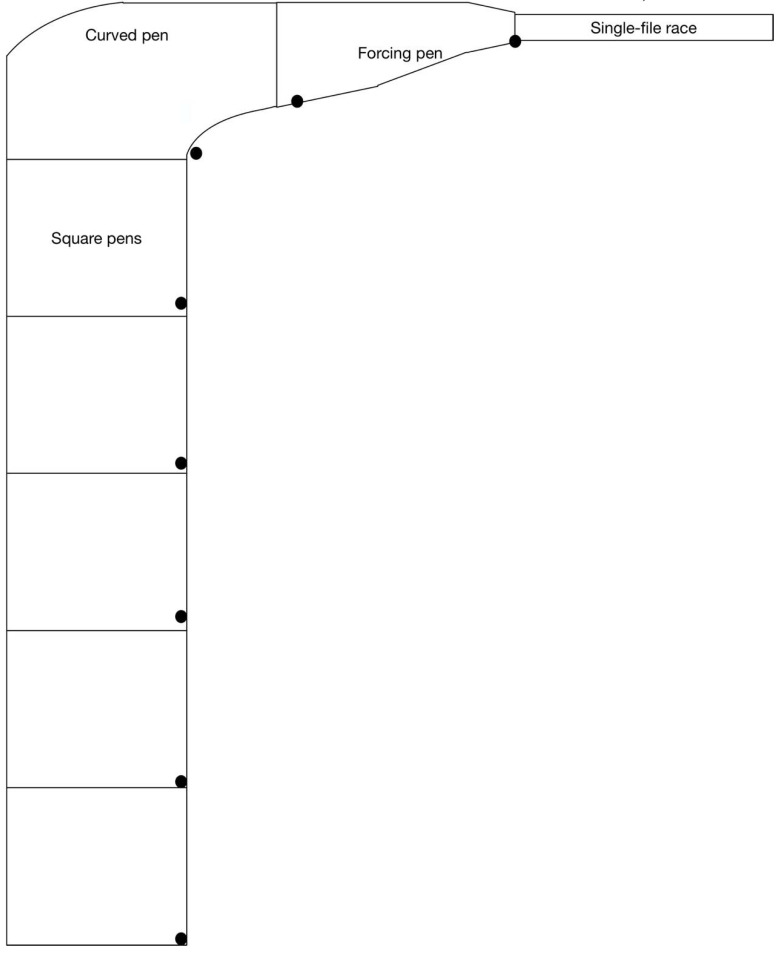
Diagram of the abattoir layout including camera locations, marked with black circles. Cameras were arranged in order from Camera 1 in the square pen at the bottom of the diagram through to Camera 6 at the curved pen, Camera 7 at the forcing pen, and Camera 8 at the single-file race.

The current pilot study examined the Optimal Flow concept by observing sheep at a large abattoir in New South Wales, Australia as they moved through a series of square pens used for lairage, then through to a curved pen that turned them through a 90-degree corner into the forcing pen and, from there, into the single-file race (see Figure 1). Dog and human interactions with sheep and with each other were also recorded, along with the rate of sheep movement (sheep per minute) through the slowest parts of the abattoir prior to slaughter. The aim of the study was to identify human and dog behaviours that correlate with stalling and stress-related behaviours in sheep. In this way, we can describe optimal flow in abattoirs more generally and provide stock handlers with information on how best to minimise distress in sheep while moving them at a steady rate that aligns with commercial imperatives. This approach permits a balance between commercial and animal welfare outcomes.

## 2. Materials and Methods

The study was conducted according to the guidelines of the Declaration of Helsinki and approved by the Ethics Committee of the University of Sydney (protocol code 2014/689, approved 20/10/2014). Informed consent was obtained from all subjects involved in the study.

### 2.1. Study Site and Camera Set-Up

The study was undertaken at an abattoir in south-west New South Wales (NSW), Australia, on 5–7 May 2015. Prior to data collection, a series of eight security cameras (Swann NHD-820 IP Cameras 1080P 25fps) was set-up to record sheep, human and dog behaviour in the last five square pens of the lairage, a curved pen, the forcing pen, and the beginning of the final single-file race that led to the knocking box. The cameras were mounted on beams that supported the roof over the pens and set to capture the majority of the pen they were mounted beside from a high angle. This allowed a view of empty space around sheep in the pen, and nearby sheep were used as a reference for how large the empty space was (in sheep-widths). The cameras were switched on via a central operating system each morning in time for processing to start at approximately 05:45 and switched off each afternoon at approximately 13:30 after processing had finished for the day. They recorded constantly throughout that time, streaming data to a hard drive (Swann NVR8-7200 1080P 3TB) via Category 5 ethernet cables.

### 2.2. Sheep

The sheep recorded during the three days of filming were from a variety of locations around NSW and had arrived by truck the day prior to filming. They occupied the lairage overnight and were recorded for the current pilot study the following morning as they were moved through the abattoir. The sheep (*n* = 3235) were of various ages, but most were meat lambs of unknown breed or cross. Sheep were penned in lairage in groups of varying size and moved through the abattoir in those groups. Sometimes sheep groups were combined with those of adjacent pens and sometimes split and sometimes kept constant. This may relate to ease of movement for stock handlers or keeping sheep from the same origin together.

### 2.3. Humans and Dogs

Five human stock handlers and seven stock dogs were filmed interacting with the sheep over the three-day study period. The same humans were present each day, but occasionally varied in their position along the races. The seven dogs recorded over the three-day period were present on some days and not others. Dog teams were rotated in and out so that they shared eight-hour shifts. The dogs were Australian kelpies or kelpie mixes of both sexes. They wore muzzles at all times while working with sheep. One dog was a puppy and was held by a stock handler on a leash when around the sheep.

### 2.4. Video Sampling

Videos were sampled across the 24 h of filming. Three start times for each of the three days of filming were selected using a random time generator available online [7] to ensure that the selection of sheep groups to follow was not influenced by the researchers. The time generator was set to a time range that included all sheep groups that moved from Camera 1 through to Camera 8 during that day. Groups of sheep were chosen rather than individual sheep because it was impossible to keep track of a single sheep within a group of sheep that were visually extremely similar to each other and were prone to sudden bunching together in response to human or dog movement nearby. The next group of sheep to pass the first camera (square pen #1 in Figure 1), after the random time given by the generator, was followed through each pen to the single-file race. The number of sheep at the start of each video and the number of sheep that left a pen in each wave of movement were both recorded throughout the coding period. It was common for stock handlers to amalgamate groups of sheep in the curved or forcing pens so, on occasions, it was difficult to follow a single focal group through the entire plant. In these cases, the timestamps on the video files were used to ensure continuity between cameras, and the coding process ended when the count of sheep clearing the pen equalled the number of sheep present in the pen at Camera 1 when the focal group was selected. Where possible, the last sheep in the focal group were identified and followed in amalgamated groups, with the coding process ending once those sheep had passed Camera 8. This sometimes resulted in more sheep being included in analysis than were in the original focus group. There were instances where the group of sheep was split and some sheep were moved into the next pen while others were held in the current pen. In these cases, the remaining sheep in the pen would be observed as well as the sheep that had been moved to the next pen.

### 2.5. Video Coding

Dog, human and sheep ethograms were developed based on previous relevant studies [8] and used to record behaviour of interest. These ethograms are shown in Table 1, Table 2 and Table 3. The behaviours recorded included dog and human behaviours that may directly influence sheep movement as well as sheep behaviour that may indicate distress [8]. These behaviours in all three species were coded with frame-by-frame analysis using the coding software The Observer v12 (Noldus Information Technology, Wageningen, The Netherlands), where behaviours from the ethogram are recorded in sequence with time stamps when observed in video recordings. Behaviours can be recorded as either event behaviours or state behaviours. Event behaviours were recorded every time any sheep in the group, or individual dog or human, performed them. State behaviours had a start and stop recorded, so included a duration. Sate behaviours were recorded for individuals for dogs and humans, but were recorded at a group level for groups of sheep, with one record that was started when the first sheep in the group began the behaviour and stopped when the last sheep in the group moved off camera or ceased the behaviour. See Table 1, Table 2 and Table 3 for further detail.

Sheep use of space (Density 2–6 in Table 1, where Density 1 = no sheep) was also recorded as an ordinal variable so that behaviours and Clearing could be examined in relation to how sheep were using space in the pen at the time. This was considered important in understanding how sheep bunching behaviour at a group level related to distress behaviours and how quickly sheep were moving, as this can be a visual indicator of the Optimal Flow concept.

### 2.6. Statistical Analysis

Data were analysed in R v3.3 (R Foundation for Statistical Computing) and The Observer v12 (Noldus Information Technology, Wageningen, The Netherlands). Behaviours were pooled into categories for analysis based on their presumed function to improve statistical power, as many behaviours occurred rarely. This categorisation is shown in Table 1, Table 2 and Table 3. The Observer was used to produce graphs to represent space used by dogs and sheep and associated frequency of distress behaviours in sheep by tallying counts of behaviours across all recordings on a per minute basis. Plots of predicted results from the regression models were created using the ggplot2 [9] and effects packages [10] in R, with the predicted responses extracted with the predict() function [11].

A generalised linear mixed model (lmer function from library lme4 [12]) was used to analyse the data with Log (Clearance) as the response variable. Clearance refers to the rate at which sheep can be actively moved from one camera (pen) to the next. Clearance was calculated by dividing the number of sheep that cleared the pen by the number of seconds it took for those same sheep as a group to finish leaving one pen and thereby enter the next and then multiplying by 60 to give a rate/minute measure. Sheep were recorded at all times as ‘stationary’—when all gates were closed—or ‘clearing pen’—when a gate is opened. As such, the time used to calculate Clearance was in most cases simply the ‘clearing pen’ duration.

Clearance was scaled via log transform, as there were large fluctuations in records. It was recorded as 0 when sheep were stationary, and Clearance therefore varied between 0, very high rates (300+) past some cameras (notably cameras 1–4), and very low rates (<40) (past cameras 6, 7, and 8). For this analysis, records where Clearance = 0 were excluded, as there were extensive periods where sheep were not being moved that would lead model results to be skewed towards behaviours and densities that were common when sheep were stationary. It was anticipated that this treatment of the raw data would give a good indication of optimal flow.

The model was built using a stepwise approach, with the model of best fit being determined by the AIC. The final model included Density (sheep use of space—an ordinal variable), Behaviour category, and Number start (the number of sheep in the group). Camera was added as a random effect to account for clustering of results where bottlenecks occurred in the abattoir. An interaction term of Density and Number start was also included.

A second model was built to examine behaviour when sheep were stationary or being moved, using Behaviour Category as the response variable. This model was a multinomial linear regression, using the multinom() function from the nnet package in R. This package does not enable the inclusion of random effects, so a time offset was used with Behaviour Category. This is an accepted substitution to using mixed effects [13]. This model was also built, using a stepwise approach as above. The final model included the variables Density, Behaviour Category (time offset), Number start, Camera, Clearance, and an additional variable—Stationary/moving, to include information on whether the sheep were stationary or moving at the time of the record. Two interaction terms were also included in the model—Density × Camera and Density × Clearance.

## 3. Results

**Figure 2:**
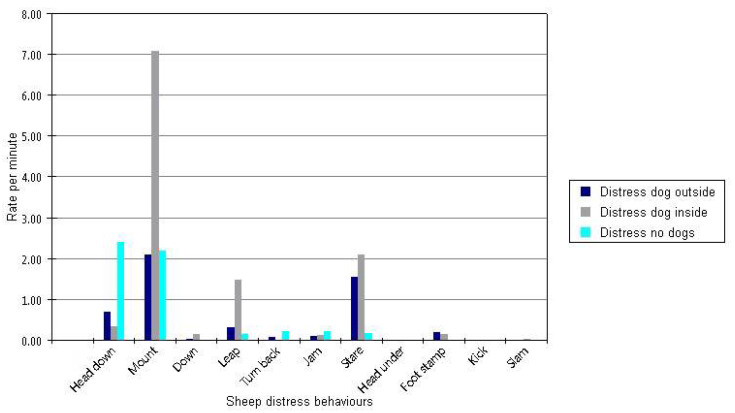
Untransformed rate of distress behaviours in sheep illustrating large fluctuations in commonality. Number of times the behaviour was recorded per minute across all video recordings analysed is shown on the *Y*-axis. Dark blue = when dogs present but outside flight zone; grey = dogs present and inside flight zone; light blue = dogs absent.

‘Distress’ behaviours among sheep were considered any of the following: head down, mount, down, leap, turn back, jam, stare, head under, kick, and slam. Clearly, some of these behaviours may be better indicators of distress than others. Head down, mount, leap, and stare were the most commonly recorded, but head down occurred more often in the absence of dogs than in the presence of dogs (Figure 2). Mount, leap and stare all occurred most often when dogs were within the flight zone (one sheep-body-length), but mount occurred at comparable frequencies when dogs were outside the flight zone and when dogs were absent. Kick, foot stamp, and down were behaviours that occurred at very low frequencies, but typically in the presence of dogs. Turn back and jam were specific to the forcing pen and single-file race, and occurred at similar frequencies both when dogs were present and when dogs were absent.

**Figure 3:**
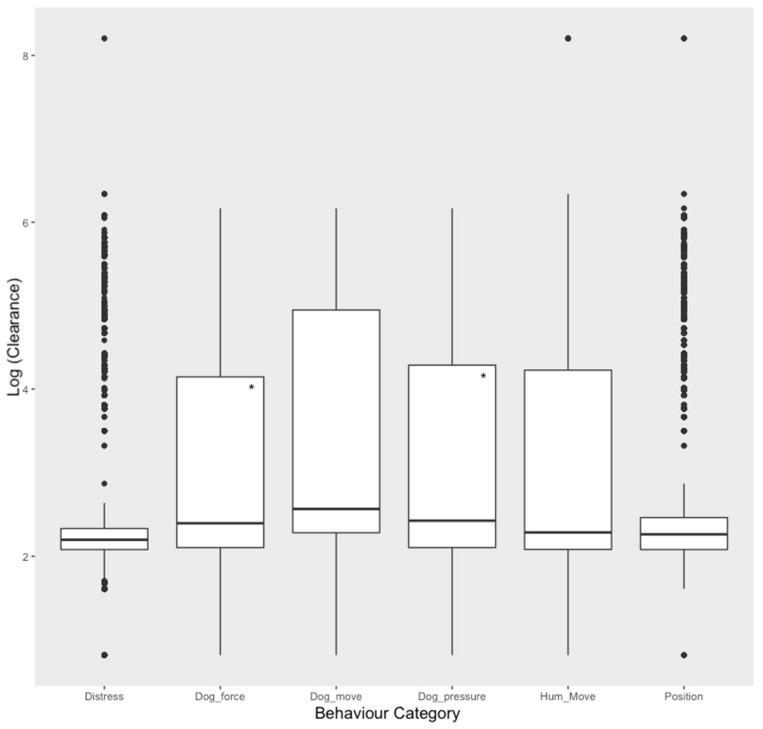
Predicted relationship between behaviour categories and Log (Clearance). Statistically significant results marked with an ‘*’.

The results of the generalised linear mixed model showed that rate of movement of sheep was significantly associated with some behaviour categories (see Figure 3). Dog force and dog pressure had a negative association with rate of sheep movement (Clearance) (Estimate = −0.11, S.E. = 0.03, *t*-Value = −4.32 and Estimate = −0.11, S.E. = 0.02, *t*-Value = −4.75, respectively).

**Figure 4:**
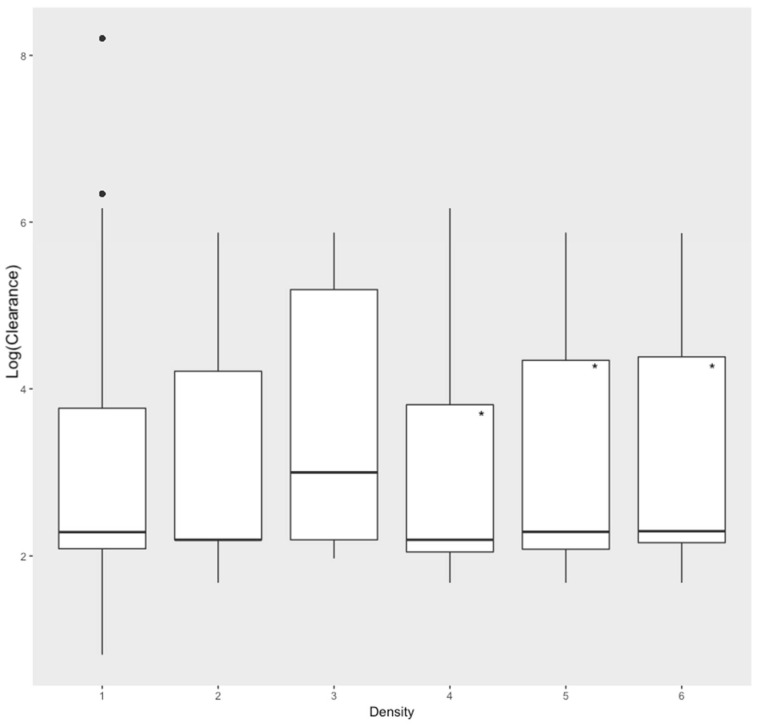
Predicted Log (Clearance) of sheep at different sheep densities (reference level is Density = 2). Density refers to sheep use of space. 1 = no sheep, 2 = free, 3 = loose, 4 = moderate, 5 = bunched, 6 = packed. Statistically significant results marked with an ‘*’.

The number of sheep in a pen at the start of recording had a small but significant, negative association with rate of sheep movement (Estimate < −0.01, S.E. < 0.01, *t*-Value = −2.61). Rate of movement increased with sheep densities that were moderate (4), bunched (5) and packed (6) (Estimate = 0.17, S.E. = 0.05, *t*-Value = 3.87; Estimate = 0.10, S.E. = 0.04, *t*-Value = 2.17 and Estimate = 0.22, S.E. = 0.05, *t*-Value = 4.72 respectively), as shown in Figure 4.

There were very small, but significant effects rate of sheep movement from sheep density and number of sheep interactions (see Table 4). Finally, as expected, there were significant, positive effects of movement and space use (analysed by the additional factor of Density) by sheep on rate of movement (see Table 4).

The multinomial regression model analysed the effect of factors on behaviour categories. This model contained interaction terms and thus the results are extensive. They are shown in full in Table S1 (Supplementary Materials). Selected results of factors of particular interest are shown in Table 5.

**Figure 5:**
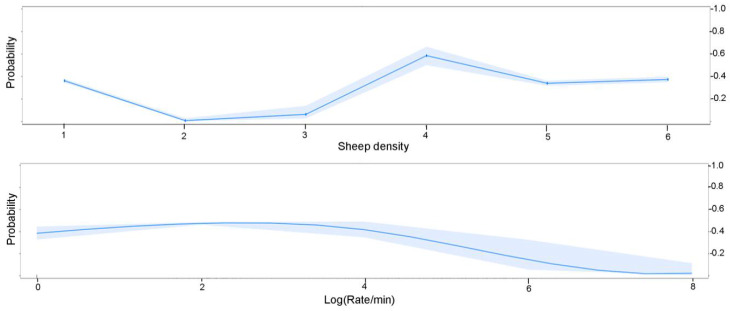
Probabilities extracted from the regression models showing the relationship between the probability of sheep distress behaviours being expressed and sheep density on the top and log (Rate/min) sheep movement speed on the bottom. Distress behaviour probability peaks at ‘moderate’ density and at moderate speed.

The factors that had strong and statistically significant effects on the likelihood of distress behaviours being expressed by sheep were sheep density and rate of movement. Figure 5 shows the probability of sheep distress behaviours being expressed given sheep density and rate of movement. Distress behaviours were significantly less likely at all densities compared to the reference level, ‘free’ (see Table 5), except in the case of moderate density, where distress behaviours increased (Estimate = 19.61, S.E. = 0.34, *p*-Value < 0.01). An interaction term between density and rate of movement was added to the model and produced very small, but statistically significant, effects all of which were associated with a slight increase in the likelihood of sheep distress behaviours. Distress behaviours of sheep decreased as rate of movement increased (Estimate = −3.19, S.E. = 0.18, *p*-Value < 0.01), which is also illustrated in Figure 5.

Dog pressure was most strongly associated with moderate sheep density (Estimate = 19.63, S.E. = 0.13, *p*-Value < 0.01), but was significantly more likely at all densities compared to the reference density of free. Conversely, in the case of the interaction term of density × rate, dog pressure effects were smaller and less likely when density was moderate than when density was free (Estimate = −14.65, S.E. = 0.25, *p*-Value < 0.01). Dog force was more likely at all densities than it was at free density except for moderate density (see Table 5). The interaction term was reversed, showing dog force positively associated with density × rate (Estimate = 10.82, S.E. = 0.22, *p*-Value < 0.01), but negatively influenced at all other levels of density in the interaction term (Table 5).

## 4. Discussion

This pilot study has revealed that ovine responses that are probably indicative of stress increased in frequency as sheep bunched more tightly together, peaking at moderate density but remaining elevated when sheep were bunched and packed. This aligns with the treatment of tight flocking behaviour as an anti-predator response in the literature [14]. However, some responses, such as head down, are difficult to perform when sheep are at high density. Head down has been identified in previous research as a behaviour indicative of stress in sheep at abattoirs [8]. Yet, there is little space available for the sheep to drop their heads when they are closely bunched, and to do so may make them vulnerable to injury if they are pushed forward by other sheep crowding close behind. Other behaviours may serve dual purposes at high densities. For example, mounting has been considered an escape behaviour [15], but may also give sheep more visual information in times of threat, relieve physical pressure from surrounding sheep, and allow access to cooler air above the flock. Furthermore, some behaviours (staring, foot stamp, kick, slam) occurred only when dogs or humans are present and are thus most likely to emerge in response to specific behaviours from handlers or dogs.

The context-specificity of likely distress behaviours has been raised before [15], but this may not be a barrier to the recognition of a given behaviour as an indicator of distress [16]. Head down when sheep are under pressure may indeed be an indicator of distress. Conversely, head down occurred frequently when no handlers or dogs are present but when sheep had been left stationary for several minutes or more. However, head down when the sheep are stationary may reflect behavioural needs other than coping, a function that is not necessarily related to distress. For example, sheep may lower their heads to investigate the ground for signs of food. A good behavioural indicator of distress should occur only when the animal is distressed, and not occur in other contexts [17]. Distress behaviours were most probable at lower rates of sheep movement. This may be indicative of distressed sheep moving more slowly or it may be that, in sheep that are moving slowly such as when moving through bottlenecks, more pressure is used to keep them moving as fast as possible, and they consequently express more distress behaviours. It is not known whether such pressure would be necessary to maintain steady sheep movement, because there was little variation in density as sheep moved through bottlenecks. This lack of variation may be particularly true of the forcing pen (Camera 7) and single-file race (Camera 8) where the movement of sheep is limited by the need to funnel them into single-file. Such bottlenecks may represent areas where pressure applied to sheep has limited effect on the speed they move. However, caution is required in this interpretation, as different abattoirs have different workplace practices and also different design flaws. It may be that it is necessary for sheep to be pressured to move past particular obstacles, or that the stock handlers believe it to be necessary. In either case, if no attempts are made to move sheep at lower densities, then it cannot be determined if pressuring sheep is required and how much. Further studies of different abattoirs with different common practices in sheep handling are recommended to reveal the true effect, if any, of sheep density on rate of movement.

The current findings reveal a positive relationship between Density and Clearance at all sheep densities when compared to the reference level, Free (2), but this relationship does not hold for the density category, Loose, which is associated with a higher rate of movement than other densities. This finding may be due to the frequent habit of moving sheep through more than one pen at a time. Once the sheep start to move from one pen to the next, their momentum can carry them through several empty pens at a fast pace, but they tend to maintain more space around them when moving freely.

Dog presence had a negative effect on Clearance. Dog use may be most closely associated with higher sheep density, but this does not necessarily reflect a causal relationship. Dogs are most likely to be used around bottlenecks in the abattoir where the rate of movement inevitably drops. In the abattoir in this study, dogs were primarily used to maintain pressure on sheep in the curved pen as they approached the forcing pen. Although it is not established whether these canine behaviours cause bunching, it would be a likely outcome if bunching is an ovine response to predators, as suggested by Hansen et al. [14].

Several interaction terms were included in models as they improved model fit. The interaction term of Density × Number_start had very small effects in the model examining Clearance, but the effects of the interaction term Density × Clearance on Behaviour category was stronger, especially for Loose density. These results suggest that the rate of sheep movement and the way sheep use space are inter-related, which is expected, but the degree to which they are inter-related varies. Sheep were frequently moved at the Moderate (4) density, but perhaps at Loose density they are most able to express distress behaviours.

The existence of bottlenecks in the route of sheep travelling through the abattoir places a strong emphasis on what occurs at those bottlenecks to the exclusion of other parts of the route. It is true that rate of sheep movement from the beginning of the route to the end is, in large part, dictated by the ease with which sheep are moved through those bottlenecks. However, concentrating data collection at bottlenecks will result in sampling only at points where sheep are likely to be under constant pressure and clustering together which, as shown in the current study, does not necessarily allow for the expression of stress behaviours. Collecting data at parts of the route through the abattoir where sheep are at rest for extended periods or moved under less pressure provides an opportunity to record sheep behaviour under different circumstances than those at bottlenecks, and thus provides a useful comparison in the quest for identifying how sheep should be moved through abattoirs to minimise stress and maximise efficiency.

### Future Research

The difficulty in analysing the data in this pilot study highlights the complex nature of applied animal behaviour where multiple species are involved. Sheep are likely to behave differently depending on how dogs and humans in the system also behave, but this is difficult to quantify at a population level. An added complication that was not foreseen was the lack of variation in sheep density at specific points in the abattoir chain. Where resources are available, this could be addressed by collecting data at other abattoirs with different cultures and practices that relate to how dogs and sheep are handled.

Further work is needed to establish if head down behaviour in contexts where sheep are stationary and unpressured is associated with established indicators of high arousal. Further study into where among flocks of sheep different putative distress behaviours occur most often may reveal their purpose of these responses and allow an exploration of their value as indicators of current welfare state. Head down is currently considered an indicator of sheep distress but, sometimes, this behaviour may not even be possible for sheep to display and may occur for different reasons at other times.

Future studies should examine factors that might motivate handler behaviour. For example, a perceived lack of control of events has been positively associated with forceful handling procedures, such as shouting and hitting [18]. Consequently, behavioural observations in abattoirs paired with attitudinal questionnaires may provide insights about how best to discourage handler behaviours that might compromise sheep (and dog) welfare and impede optimal flow.

## 5. Conclusions

This pilot study of triadic interactions between humans, dogs and sheep in an abattoir indicates that, where possible, Optimal Flow emerges when sheep density is categorised as Free or Loose. This is where the rate of sheep clearance from pens is high but frequency of distress behaviours in sheep is low. However, this may be difficult to achieve in parts of abattoirs where bottlenecks occur and flaws in abattoir design may discourage sheep from moving forward of their own volition. Maintaining sheep density in these circumstances at a Moderate state provides a balance between distress behaviour frequency and Clearance A density of Bunched or Packed sheep is associated with a decrease in Clearance. This study showed a decrease also in distress behaviours at those higher densities, but this may not be absolutely indicative of lower sheep stress, but rather a decreased ability for sheep to express distress. Abattoirs should consider the necessity of maintaining higher densities. If sheep can be sufficiently motivated to move at low density, this should be considered to align with the Optimal Flow concept. Dog use at the current abattoir was associated with increased distress behaviours among sheep.

## Figures and Tables

**Table 1:** Sheep ethogram.

Behaviour	Description	Type	Rationale for Collecting These Data	Category
Head Down	Sheep lowers head so its eyes are below level of the point of the scapula.	Event	Hemsworth et al. (2011) show that lowering the head below this point was significantly positively correlated with elevated cortisol.	Distress
Mount	Both front legs come off the ground and at least one is placed on top of another sheep.	Event	Probably occurs when sheep are under inescapable pressure.	Distress
Down	Sheep is in either lateral, ventral or dorsal recumbency.	Event	Often occurs in non-resting sheep when they are bunched very tightly. Risk of injury, bruises, stress.	Distress
Leap	All four feet are off the ground and the sheep is momentarily airborne.	Event	Escape behaviour, associated with startling or feature of ground surface that the sheep aims to avoid (e.g., high-contrast flooring).	Distress
Circling	Majority of the sheep in the pen are in physical contact with another sheep and at least some of the group is moving in either a clockwise or anti-clockwise direction, with moving sheep consistently turning in towards centre of group. Each moving sheep is in contact with another moving sheep, forming a circle.	State	Bunching may be comforting to sheep but, if they start to move away from a stimulus, they may fail to get far enough away from it for comfort. In the absence of consummation, the circular movement may become relentless.	Distress
Staring	At least one sheep’s gaze is fixed on a dog or human. Sheep may glance away, but for only an instant before fixating again. One record for every occurrence on camera. Duration is measured by starting when the first sheep’s gaze lasts for longer than a second and ending when the last sheep looks away for more than a second.	State	Vigilance towards a particular stimulus, probably indicating that stimulus is threatening or interesting.	Distress
Turn Back	Sheep at the front of the mob turn around to face the back of the mob and move against the flow.	Event	This reflects poor facility design because it primarily occurs when sheep see no way forward, or when something in front of them disquiets them (Grandin, 1990).	Distress
Jam	In opening to single-file race or within the single-file race itself, where sheep are not moving forward or backward because two or more individuals are jammed together against the sides of the race.	Event	A jam decelerates the flow, and may cause sheep behind to turn back. Sometimes direct human or canine intervention is required, so sheep are touched or pushed.	Distress
Empty Race	Applicable only to single-file race, where the last sheep’s hindquarters remain visible at the top of the frame, but no sheep can be seen at the bottom of the frame.	State	Reveals interrupted flow in the single-file race, primarily because of a jam at the forcing pen.	Distress
Head Under	One sheep has lowered its head and then moved forward so its head is under the ventrum of another sheep. Indicated by one sheep being lifted off front or hind feet by the sheep underneath. May start as head down, but considered separate behaviour where there is a pause of at least 1-s between head down and the sheep moving forwards to the head under position.	Event	Similar to head down, but the sheep has also moved forward while in close proximity to other sheep.	Distress
Foot stamp	Sheep lifts one front foot and brings it down to the ground forcefully in the same place, without moving other feet. One record for each occurrence.	Event	This is considered a defensive behaviour, usually in the presence of predators (Hansen et al., 2001).	Distress
Slam	Running sheep makes sufficient impact with infrastructure that they rebound off it. One record for every occurrence	Event	This may bruise carcasses (most commonly at the flank or on the chest), and is typically seen when sheep are fleeing from a dog.	Distress
Back up in Race	Applicable only to single-file race, where at least one sheep in frame reverses with both front and hindfeet so that at least one full stride is taken backwards.	Event	Amounts to disrupted flow in single-file race, either as a result of a stock person moving ahead of or alongside sheep.	Distress
Kick	Sheep hindfoot strikes out behind, with associated leg fully extended, and makes contact with dog, human or another sheep. One record for every	Event	Recorded rarely, but presumed to be an offensive behaviour coupled with escape.	Distress
Clearing Pen	From when gate is opened until either pen is clear of sheep or gate is closed again.	State	This provides a measure of the latency to clear a pen.	Movement
Stationary	Sheep are waiting in a pen and not being actively moved to the next pen or stage. Started when all gates are closed after sheep have entered a new pen. Stopped when gate is opened immediately prior to sheep moving to a new pen.	State	This provides a measure of the duration of sheep as they idle in a pen before being moved, and whether this rest period affects how easily they can subsequently be moved.	Movement
Density 2 Free	Sheep occupation of space is such that there is more than one sheep-body-width of empty space in each direction surrounding the majority of the sheep in the group.	State	-	Space
Density 3 Loose	Sheep occupation of space is such that there is approximately one sheep-body-width in two directions surrounding the majority of the sheep in the group.	State	-	Space
Density 4 Moderate	Less than one sheep-body-width in at least one direction surrounding the majority of sheep in the group, but sufficient empty space around the sheep to allow sideways or forward movements that create the space for a single sheep in the group to pass between two other sheep in the group.	State	-	Space
Density 5 Bunched	No visible empty space between sheep flanks, but empty space is visible in front or behind of the majority of the sheep in the group.	State	-	Space
Density 6 Packed	No empty space visible in any direction surrounding the majority of the sheep in the group.	State	-	Space

**Table 2:** Dog ethogram.

Behaviour	Description	Type	Rationale	Category
Rush	Dog rushes from outside flight zone * for a distance of at least one sheep-body-length towards sheep and may snap or jump at them.	Event	Dogs that do this are penetrating well into the sheep’s flight zone and sheep may not have the opportunity to escape from them.	Force
Parked	One or more dog is stationary in same pen as sheep. Dog may make adjustments in position involving less than two sheep-body-lengths. Recorded as duration.	State	The stationed dog is passive, but puts pressure on sheep by its presence. If the dog is stationed too close, the sheep may mount or turn to face the dog.	Pressure
Back	Dog jumps onto and travels over backs of sheep.	Event	-	Pressure
Walking	Dog is walking, defined as a four-beat gait. Recorded as duration.	State	-	Dog movement
Stalking	Dog is stalking, with the body lowered, head up or lowered and extended forward, ears erect, on top of head and pointing forward, tail below back level and motionless. Forward motion is slow to medium in a four-beat gait. Recorded as duration.	State	-	Dog movement
Trotting	Dog is trotting, defined as a two-beat gait. Recorded as duration.	State	-	Dog movement
Canter	Dog is cantering, defined as a three-beat gait. Recorded as duration.	State	-	Dog movement
In flight zone	Dog is within one sheep-body-length of sheep. Recorded as duration.	State	-	Force
Outside of flight zone	Dog more than one sheep-body-length from sheep. Recorded as duration.	State	-	Pressure
Gaze	Dog is looking in fixed direction.	State	Gaze direction indicates what the dog is most likely to be responding to.	Human-dog interaction
Physical contact	Dog’s head, mouth or sternum makes physical contact with sheep.	Event	-	Force

* The flight zone was one sheep-body-length.

**Table 3:** Human ethogram.

**Behaviour**	**Modifiers**	**Description**	**Type**	**Category**
Distance from sheep	Outside of flight zone	Handler is further than one sheep-body-length from any sheep present.	State	Position
Distance from sheep	Inside of flight zone	Handler is within one sheep-body-length of any sheep present.	State	Position
Position along race/mob	Near head of mob	Handler is within one sheep-body-length of the sheep at the front of the mob.	State	Position
-	Near rear of mob	Handler is within one sheep-body-length of the sheep at the rear of the mob (sheep farthest from intended direction).	State	Position
Movement	Same direction as mob	Handler is moving in the same direction as sheep.	State	Human movement
-	Neutral movement	Handler is moving but neither in the same or opposite direction as sheep.	State	Human movement
-	Opposite direction as sheep	Handler is moving in the opposite direction to sheep movement.	State	Human movement
Arm position	Forearms protracted	Handler has forearms above hipline, upper arms by sides (including arms folded).	State	Human movement
-	Whole arm protracted	Handler has both upper and lower arm raised or outstretched.	State	Human movement
Arm movement	One arm moving	Handler is moving one arm.	Event	Human movement
-	Both arms moving	Handler is moving both arms.	Event	Human movement
Plastic bag/bell use	Held but not in use	Handler has a plastic bag or bell but is not shaking the object.	Event	Object interaction
-	In use	Handler has a plastic bag or bell and is shaking the object.	Event	Object interaction
Gaze direction	Sheep	Time handler spends with face and eyes (if visible) pointing directly at sheep.	State	Object interaction
-	Dog	Time handler spends with face and eyes (if visible) pointing directly at dog.	State	Object interaction
Gate manipulation	Handler opens gate—no jamming	Handler opens a gate within or between races, without physically pushing against sheep.	Event	Object interaction
-	Handler opens gate—jamming	Handler opens a gate within or between races, gate is physically pushed against one or more sheep.	Event	Object interaction
-	Handler shuts gate—no jamming	Handler shuts a gate within or between races without physically pushing against sheep.	Event	Object interaction
-	Handler shuts gate—jamming	Handler shuts a gate within or between races, gate is physically pushed against one or more sheep.	Event	Object interaction
Foot touch	-	Handler touches sheep with foot.	Event	Animal directed
Touching sheep	-	Handler touches a sheep with one hand.	Event	Animal directed
Grabbing sheep	-	Handler grabs or touches a sheep with both hands.	Event	Animal directed
Touching dog	-	Handler pats, rubs or scratches dog.	Event	Animal directed

**Table 4:** Selected results of the linear mixed model examining factors affecting the rate of sheep movement per minute, showing only the statistically significant results. Reference level is Density = 2. Std Error is the Standard Error of the Estimate (S.E.). “*” represents statistically significant results.

Factor	Estimate	Standard Error	*t*-Value
(Intercept)	3.83	0.52	7.43
Dog_force	−0.11	0.03	−4.32
Dog_pressure	−0.11	0.02	−4.75
Sheep_movement	0.13	0.04	3.44
Space	0.14	0.02	5.89
Moderate (Density4)	0.17	0.05	3.87
Bunched (Density5)	0.10	0.04	2.17
Packed (Density6)	0.22	0.05	4.72
Number_start	<0.01	<0.01 *	−2.61
Density1: Num_start	0.01	<0.01 *	4.19
Density3: Num_start	0.01	<0.01 *	2.63
Density5: Num_start	<0.01 *	<0.01 *	3.11
Density6: Num_start	<0.01 *	<0.01 *	3.79

**Table 5:** Selected results from the multinomial model examining factors influencing behaviour category. See Table S1 for full results. Reference category is Density = 2 (free). “*” represents statistically significant results.

Factor	Distress	Dog Force	Dog Move	Dog Pressure	Human-Dog	Human Move	Movement
(Intercept)	17.98	−9.45	−4.39	−5.60	−12.63	15.66	7.79
SE	0.37	0.46	0.46	0.48	0.52	0.29	0.60
*p*-Value	<0.01 *	<0.01 *	<0.01 *	<0.01 *	<0.01 *	<0.01 *	<0.01 *
Clearance	−3.19	3.20	2.09	−1.45	−6.72	−1.89	−9.71
SE	0.18	0.20	0.19	0.23	0.11	0.14	0.23
*p*-Value	<0.01 *	<0.01 *	<0.01 *	<0.01 *	<0.01 *	<0.01 *	<0.01 *
Density1	−15.80	9.35	3.24	5.92	10.61	−13.75	−9.31
SE	0.36	0.53	0.51	0.48	0.48	0.27	0.47
*p*-Value	<0.01 *	<0.01 *	<0.01 *	<0.01 *	<0.01 *	<0.01 *	<0.01 *
Density3	19.61	−37.92	14.82	19.63	5.68	−4.01	−3.32
SE	0.34	0.04	0.23	0.13	<0.01	0.20	0.39
*p*-Value	<0.01 *	<0.01 *	<0.01 *	<0.01 *	<0.01 *	<0.01 *	<0.01 *
Density4	−18.10	0.75	−1.78	2.81	−9.58	−16.11	−13.15
SE	0.42	0.70	0.82	0.85	<0.01	0.40	0.53
*p*-Value	<0.01 *	0.29	0.03	<0.01 *	<0.01 *	<0.01 *	<0.01 *
Density5	−17.12	9.11	3.65	4.13	−6.56	−15.50	−10.48
SE	0.39	0.57	0.52	0.36	0.43	0.35	0.53
*p*-Value	<0.01 *	<0.01 *	<0.01 *	<0.01 *	<0.01 *	<0.01 *	<0.01 *
Density6	−15.53	10.38	−5.36	6.02	−0.51	−13.58	−31.72
SE	0.40	0.44	0.32	0.40	0.41	0.34	0.45
*p*-Value	<0.01 *	<0.01 *	<0.01 *	<0.01 *	0.21	<0.01 *	<0.01 *
Num_start	<0.01	<0.01	<0.01	<0.01	<0.01	<0.01	−0.01
SE	<0.01	<0.01	<0.01	<0.01	<0.01	<0.01	<0.01
*p*-Value	0.30	0.81	0.24	0.28	0.11	0.04	<0.01 *
Clearance × Density1	2.43	−4.01	−2.56	0.60	0.97	1.72	9.87
SE	0.18	0.19	0.16	0.21	0.12	0.14	0.22
*p*-Value	<0.01 *	<0.01 *	<0.01 *	<0.01 *	<0.01 *	<0.01 *	<0.01 *
Clearance × Density3	4.89	10.82	0.02	−14.65	7.23	4.75	11.85
SE	0.18	0.22	0.20	0.25	<0.01*	0.18	0.27
*p*-Value	<0.01 *	<0.01 *	0.93	<0.01 *	<0.01 *	<0.01 *	<0.01 *
Clearance × Density4	2.94	−2.14	−1.28	1.57	−1.13	2.12	10.44
SE	0.20	0.26	0.24	0.28	<0.01 *	0.16	0.24
*p*-Value	<0.01 *	<0.01 *	<0.01 *	<0.01 *	<0.01 *	<0.01 *	<0.01 *
Clearance × Density5	2.74	−3.56	−2.28	1.00	1.68	2.04	10.22
SE	0.19	0.20	0.17	0.21	0.15	0.15	0.22
*p*-Value	<0.01 *	<0.01 *	<0.01 *	<0.01 *	<0.01 *	<0.01 *	<0.01 *
Clearance × Density6	2.80	−3.68	−2.45	0.95	0.99	1.77	9.96
SE	0.18	0.20	0.17	0.22	0.15	0.15	0.22
*p*-Value	<0.01 *	<0.01 *	<0.01 *	<0.01 *	<0.01 *	<0.01 *	<0.01 *

## Data Availability

The data presented in this study can be obtained on request from the corresponding author.

## Supplementary Materials

The following are available online at https://www.mdpi.com/2076-2615/11/2/344/s1, Table S1: Complete statistical output of the multinomial model examining factors affecting behaviour categories observed.
