# Supplementary material for: Optimal Flow—A Pilot Study Balancing Sheep Movement and Welfare in Abattoirs
Source: Animals (Basel). 2021 Jan 29;11(2):344. doi: 10.3390/ani11020344 (PMC7912567; doi:10.3390/ani11020344)
Supplement: Supplementary file 1 [file animals-11-00344-s001.pdf]

**Estimate**	Distress	Dog_force	Dog_move	Dog_pressure	Hum_Dog	Hum_Move	Movement	Obj_Int	Position	Space
(Intercept)	17.98	-9.45	-4.39	-5.60	-12.63	15.66	7.79	-2.56	1.32	22.54
Rate	-3.19	3.20	2.09	-1.45	-6.72	-1.89	-9.71	2.14	1.34	-3.66
Density1	-15.80	9.35	3.24	5.92	10.61	-13.75	-9.31	2.66	-1.08	-24.43
Density3	19.61	-37.92	14.82	19.63	5.68	-4.01	-3.32	-36.01	29.08	15.39
Density4	-18.10	0.75	-1.78	2.81	-9.58	-16.11	-13.15	-4.71	-3.24	-23.79
Density5	-17.12	9.11	3.65	4.13	-6.56	-15.50	-10.48	0.92	-2.28	-22.33
Density6	-15.53	10.38	-5.36	6.02	-0.51	-13.58	-31.72	3.41	-0.25	-20.58
Num_start	0.00	0.00	0.00	0.00	0.00	0.00	-0.01	-0.01	0.00	-0.01
Beh_cat.1Distress	1.14	1.31	1.05	1.32	0.92	0.46	0.22	0.71	0.61	0.82
Beh_cat.1Dog_force	1.55	2.41	1.84	2.03	1.95	0.67	0.79	-0.47	1.14	1.14
Beh_cat.1Dog_move	1.08	2.14	1.81	1.97	2.34	0.68	0.51	0.93	1.02	1.05
Beh_cat.1Dog_pressure	1.17	2.11	1.63	1.95	1.57	0.55	0.48	0.45	0.58	0.83
Beh_cat.1Hum_Dog	0.71	1.64	1.95	1.79	2.86	1.24	-0.57	0.57	1.36	0.73
Beh_cat.1Hum_Move	0.23	0.92	0.84	0.86	1.41	0.30	-0.16	0.18	0.31	0.11
Beh_cat.1Movement	1.31	1.65	1.39	1.58	1.82	0.93	-0.33	1.46	0.75	1.10
Beh_cat.1Obj_Int	0.94	1.50	1.53	1.82	2.06	1.00	1.01	1.08	1.27	0.88
Beh_cat.1Position	0.67	1.31	1.02	1.17	1.78	0.50	-0.07	0.54	0.65	0.51
Beh_cat.1Space	0.82	1.00	1.01	0.94	1.87	0.41	0.08	0.82	0.44	0.83
Camera2	-8.47	-3.55	-4.52	14.44	-10.99	-0.52	48.57	-5.26	-4.22	2.29
Camera3	26.50	5.13	4.13	-3.87	6.95	-6.73	15.47	-5.20	-3.66	24.84
Camera4	5.01	-6.60	-2.81	-4.78	-3.31	-0.16	14.28	-3.77	-0.44	0.59
Camera5	1.97	2.62	5.29	3.14	1.48	3.58	9.09	2.98	3.51	13.42
Camera6	2.66	5.64	7.93	5.34	7.21	-1.09	9.07	-0.44	-2.56	5.83
Camera7	-9.67	-11.05	-11.61	-2.75	-3.49	-7.37	30.43	-3.44	-5.50	-7.10
Camera8	-21.93	-21.54	-20.51	-21.12	-13.90	-16.11	2.00	-20.44	-8.66	-24.93
Stat_moveS	2.34	-0.83	0.39	-0.22	1.32	1.21	4.15	1.33	1.02	2.84

Rate:Density1	2.43	-4.01	-2.56	0.60	0.97	1.72	9.87	-2.46	-1.48	3.83
Rate:Density3	4.89	10.82	0.02	-14.65	7.23	4.75	11.85	13.10	1.63	5.63
Rate:Density4	2.94	-2.14	-1.28	1.57	-1.13	2.12	10.44	-1.07	-0.94	3.94
Rate:Density5	2.74	-3.56	-2.28	1.00	1.68	2.04	10.22	-2.07	-1.05	3.70
Rate:Density6	2.80	-3.68	-2.45	0.95	0.99	1.77	9.96	-2.43	-1.43	3.50
Density1:Camera2	7.65	3.14	0.36	-17.12	2.08	-2.26	-49.24	2.90	0.81	-3.39
Density3:Camera2	23.86	-8.41	-7.47	-2.06	2.10	-0.68	-3.03	-8.17	-10.10	11.80
Density4:Camera2	28.96	22.33	19.57	1.31	5.43	-9.53	-27.73	-1.83	-10.28	18.05
Density5:Camera2	8.24	2.39	0.04	-16.32	-14.89	-2.33	-49.89	1.80	0.68	-2.59
Density6:Camera2	7.53	2.47	10.07	-39.60	-6.24	-2.40	-25.77	2.33	1.11	-3.86
Density1:Camera3	-21.51	1.20	1.64	8.43	-3.29	11.49	-9.80	9.61	9.59	-20.92
Density3:Camera3	-22.21	12.26	23.38	0.69	-0.04	-1.07	20.28	-7.62	-5.45	-20.38
Density4:Camera3	-3.71	18.98	17.54	-3.27	8.45	29.04	9.01	5.36	27.04	-2.32
Density5:Camera3	-22.35	-1.37	-0.86	7.63	8.63	11.22	-10.96	9.59	8.47	-21.28
Density6:Camera3	-23.75	-2.45	7.57	-13.93	-6.59	10.12	11.18	7.55	7.18	-23.15
Density1:Camera4	-3.89	9.26	6.59	8.10	4.40	1.88	-12.78	4.80	2.83	0.76
Density3:Camera4	-4.44	-7.36	-3.87	-7.40	2.56	22.09	15.01	-15.03	2.44	-1.85
Density4:Camera4	-3.45	-21.17	-18.64	-12.81	0.15	0.66	-12.80	4.23	0.19	0.84
Density5:Camera4	-3.55	7.91	4.63	8.03	-5.77	1.35	-13.58	3.81	1.03	0.76
Density6:Camera4	-6.08	6.79	14.00	3.48	-4.10	0.51	8.86	3.44	0.61	-1.28
Density1:Camera5	-0.70	-2.98	-4.09	-3.20	-3.83	-2.69	-7.63	-2.25	-1.76	-12.92
Density3:Camera5	3.01	-3.79	-4.93	-0.88	1.57	-1.97	25.87	-6.31	-5.54	-8.70
Density4:Camera5	17.40	15.89	13.29	12.46	7.04	14.99	12.44	15.84	15.85	6.40
Density5:Camera5	0.42	-1.99	-3.25	-0.48	-9.90	-0.58	-5.75	-0.34	-0.38	-10.61
Density6:Camera5	-2.14	-4.62	4.61	-4.64	6.58	-3.12	14.75	-3.36	-2.49	-13.69
Density1:Camera6	-3.27	-3.94	-5.00	-3.59	-5.95	0.85	-9.24	-0.41	2.63	-6.57
Density3:Camera6	-5.73	-1.30	-1.27	-0.98	-0.03	0.11	-0.07	0.01	-0.20	9.48

Density4:Camera6	17.15	18.77	15.18	15.20	-3.41	-5.61	12.64	-0.36	-10.45	14.89
Density5:Camera6	-1.28	-2.78	-4.53	-1.12	11.13	2.89	-7.66	1.77	3.85	-4.35
Density6:Camera6	-4.21	-5.10	3.54	-4.17	5.46	0.67	13.40	-1.44	1.60	-7.61
Density1:Camera7	6.00	2.70	2.14	-6.32	-7.17	5.43	-32.32	0.68	4.65	-1.58
Density3:Camera7	-3.03	-3.86	18.81	44.22	-0.88	-4.55	-26.22	-0.62	-11.01	-9.56
Density4:Camera7	8.54	-5.73	4.45	-4.78	-9.99	6.85	-26.35	-4.17	6.07	1.76
Density5:Camera7	7.12	4.14	2.44	-3.64	10.38	6.54	-30.68	1.84	5.26	0.04
Density6:Camera7	4.49	1.25	9.93	-7.38	4.15	4.64	-10.58	0.57	2.94	-2.86
Density1:Camera8	11.54	8.17	5.45	2.53	-11.48	11.16	-11.55	5.58	5.47	13.99
Density3:Camera8	-0.13	0.11	0.78	1.84	-0.25	23.15	-9.64	0.62	-2.29	3.11
Density4:Camera8	13.73	13.00	9.10	3.76	6.86	12.69	-7.79	14.20	6.75	16.27
Density5:Camera8	12.17	-5.09	-0.46	2.09	3.04	11.65	-10.41	3.24	5.02	14.47
Density6:Camera8	10.91	-4.53	5.59	-7.60	0.51	11.19	12.23	-1.11	4.36	13.92
Rate:Camera2	0.20	0.40	1.09	0.87	6.82	0.55	0.12	0.58	0.61	0.21
Rate:Camera3	-0.66	-0.61	-0.33	-0.12	5.09	-0.64	-0.94	-0.52	-0.85	-0.57
Rate:Camera4	0.16	0.13	0.01	0.07	5.49	-0.04	-0.14	0.13	-0.11	-0.06
Rate:Camera5	0.09	0.61	0.45	0.66	6.06	0.04	-0.24	0.18	-0.11	-0.11
Rate:Camera6	0.62	0.16	0.12	0.23	5.56	0.30	0.16	0.55	0.27	0.39
Rate:Camera7	1.85	3.32	4.13	3.51	8.99	1.04	0.28	0.08	0.71	3.40
Rate:Camera8	5.32	-1.60	-1.77	0.98	9.18	2.81	4.24	-1.65	2.62	5.27

**Standard errors**	Distress	Dog_force	Dog_move	Dog_pressure	Hum_Dog	Hum_Move	Movement	Obj_Int	Position	Space
(Intercept)	0.37	0.46	0.46	0.48	0.52	0.29	0.60	0.51	0.38	0.43
Rate	0.18	0.20	0.19	0.23	0.11	0.14	0.23	0.20	0.16	0.16
Density1	0.36	0.53	0.51	0.48	0.48	0.27	0.47	0.41	0.32	0.45
Density3	0.34	0.04	0.23	0.13	0.00	0.20	0.39	0.04	0.21	0.34

Density4	0.42	0.70	0.82	0.85	0.00	0.40	0.53	0.13	0.48	0.44
Density5	0.39	0.57	0.52	0.36	0.43	0.35	0.53	0.54	0.40	0.41
Density6	0.40	0.44	0.32	0.40	0.41	0.34	0.45	0.54	0.41	0.42
Num_start	0.00	0.00	0.00	0.00	0.00	0.00	0.00	0.00	0.00	0.00
Beh_cat.1Distress	0.14	0.31	0.26	0.29	0.34	0.13	0.28	0.31	0.15	0.21
Beh_cat.1Dog_force	0.30	0.41	0.37	0.40	0.42	0.30	0.46	0.63	0.32	0.37
Beh_cat.1Dog_move	0.25	0.37	0.32	0.35	0.36	0.24	0.39	0.39	0.26	0.31
Beh_cat.1Dog_pressure	0.26	0.38	0.33	0.36	0.39	0.25	0.41	0.43	0.28	0.33
Beh_cat.1Hum_Dog	0.52	0.59	0.54	0.56	0.42	0.49	1.12	0.73	0.51	0.62
Beh_cat.1Hum_Move	0.13	0.31	0.25	0.29	0.31	0.12	0.27	0.30	0.14	0.21
Beh_cat.1Movement	0.39	0.55	0.49	0.52	0.67	0.38	0.67	0.53	0.42	0.45
Beh_cat.1Obj_Int	0.39	0.53	0.45	0.48	0.55	0.36	0.50	0.52	0.38	0.46
Beh_cat.1Position	0.15	0.33	0.27	0.31	0.35	0.14	0.32	0.33	0.16	0.23
Beh_cat.1Space	0.21	0.38	0.31	0.36	0.40	0.20	0.36	0.38	0.22	0.26
Camera2	0.36	0.67	0.95	0.92	0.11	0.73	0.48	0.78	1.02	0.56
Camera3	0.24	0.55	0.57	0.51	0.87	0.26	0.34	0.54	0.34	0.29
Camera4	0.39	0.44	0.41	0.36	0.57	0.27	0.39	0.58	0.44	0.40
Camera5	0.26	0.54	0.48	0.50	0.93	0.29	0.35	0.51	0.36	0.30
Camera6	0.31	0.46	0.46	0.46	0.52	0.34	0.43	0.54	0.41	0.35
Camera7	0.42	0.90	0.96	0.83	0.08	0.40	0.72	0.27	0.50	0.98
Camera8	0.45	0.00	0.00	0.00	0.00	0.53	0.87	0.00	0.61	0.47
Stat_moveS	0.33	0.45	0.37	0.39	0.54	0.33	0.50	0.52	0.38	0.37
Rate:Density1	0.18	0.19	0.16	0.21	0.12	0.14	0.22	0.18	0.15	0.16
Rate:Density3	0.18	0.22	0.20	0.25	0.00	0.18	0.27	0.21	0.22	0.13
Rate:Density4	0.20	0.26	0.24	0.28	0.00	0.16	0.24	0.22	0.17	0.17
Rate:Density5	0.19	0.20	0.17	0.21	0.15	0.15	0.22	0.19	0.16	0.16
Rate:Density6	0.18	0.20	0.17	0.22	0.15	0.15	0.22	0.19	0.15	0.16

Density1:Camera2	0.34	0.58	0.60	0.55	0.11	0.61	0.45	0.57	0.70	0.56
Density3:Camera2	0.34	0.00	NaN	0.00	0.00	0.00	0.48	0.00	0.00	0.41
Density4:Camera2	0.41	0.79	0.69	0.89	NaN	0.00	0.62	0.00	0.00	0.48
Density5:Camera2	0.46	0.72	0.66	0.57	0.00	0.71	0.75	0.92	0.79	0.60
Density6:Camera2	0.46	0.58	0.51	0.00	0.00	0.72	0.51	0.71	0.81	0.64
Density1:Camera3	0.34	0.54	0.50	0.46	0.67	0.29	0.43	0.45	0.32	0.41
Density3:Camera3	0.43	0.04	0.18	0.00	0.00	0.00	0.53	0.00	0.00	0.40
Density4:Camera3	0.29	0.60	0.63	0.00	0.00	0.29	0.44	0.00	0.34	0.31
Density5:Camera3	0.40	0.61	0.52	0.38	0.62	0.36	0.52	0.55	0.39	0.40
Density6:Camera3	0.41	0.53	0.42	0.00	0.00	0.37	0.43	0.57	0.44	0.45
Density1:Camera4	0.45	0.50	0.45	0.45	0.57	0.29	0.46	0.47	0.38	0.50
Density3:Camera4	0.57	0.00	0.00	0.00	0.00	0.17	0.69	0.00	1.00	0.59
Density4:Camera4	0.59	0.00	0.00	0.00	0.00	0.51	0.72	0.99	0.71	0.59
Density5:Camera4	0.51	0.55	0.48	0.36	0.00	0.39	0.58	0.68	0.48	0.51
Density6:Camera4	0.51	0.41	0.31	0.55	0.00	0.38	0.43	0.56	0.49	0.52
Density1:Camera5	0.32	0.52	0.47	0.49	0.73	0.30	0.40	0.44	0.32	0.40
Density3:Camera5	0.34	NaN	0.00	0.00	0.00	0.00	0.51	0.00	0.00	0.34
Density4:Camera5	0.28	0.51	0.50	0.82	0.00	0.30	0.41	0.87	0.36	0.29
Density5:Camera5	0.42	0.57	0.51	0.46	0.00	0.41	0.50	0.60	0.43	0.42
Density6:Camera5	0.40	0.42	0.33	0.43	0.58	0.39	0.38	0.58	0.44	0.42
Density1:Camera6	0.25	0.46	0.44	0.42	0.40	0.24	0.37	0.37	0.27	0.34
Density3:Camera6	0.00	NaN	0.00	0.00	NaN	NaN	0.00	NaN	0.00	0.00
Density4:Camera6	0.29	0.34	0.45	0.50	NaN	0.00	0.51	0.00	0.00	0.29
Density5:Camera6	0.42	0.57	0.52	0.39	0.40	0.42	0.57	0.60	0.45	0.43
Density6:Camera6	0.35	0.40	0.28	0.36	0.33	0.34	0.38	0.53	0.39	0.37
Density1:Camera7	0.24	0.51	0.53	0.52	0.50	0.23	0.50	0.45	0.26	0.45
Density3:Camera7	0.37	0.00	0.39	0.13	0.00	0.00	0.00	0.00	0.00	0.39

Density4:Camera7	0.48	0.00	0.72	0.76	0.00	0.49	0.66	0.00	0.54	0.59
Density5:Camera7	0.30	0.59	0.58	0.38	0.40	0.30	0.70	0.62	0.34	0.41
Density6:Camera7	0.31	0.38	0.40	0.39	0.40	0.31	0.59	0.49	0.37	0.43
Density1:Camera8	0.38	0.00	0.00	0.00	0.00	0.31	0.57	0.00	0.35	0.42
Density3:Camera8	0.43	NaN	NaN	0.00	0.00	0.33	0.00	0.00	0.36	0.37
Density4:Camera8	0.47	0.00	0.00	0.00	0.00	0.44	0.68	0.00	0.48	0.45
Density5:Camera8	0.42	0.00	0.00	0.00	0.00	0.37	0.61	0.00	0.41	0.39
Density6:Camera8	0.52	0.00	0.00	0.00	0.00	0.48	0.70	0.00	0.53	0.51
Rate:Camera2	0.10	0.18	0.24	0.25	0.15	0.11	0.13	0.19	0.18	0.11
Rate:Camera3	0.08	0.16	0.16	0.19	0.22	0.08	0.11	0.15	0.10	0.09
Rate:Camera4	0.07	0.14	0.13	0.14	0.19	0.07	0.11	0.14	0.09	0.08
Rate:Camera5	0.09	0.17	0.15	0.17	0.25	0.09	0.12	0.14	0.11	0.10
Rate:Camera6	0.15	0.19	0.18	0.20	0.17	0.15	0.19	0.20	0.17	0.16
Rate:Camera7	0.22	0.58	0.60	0.55	0.20	0.21	0.56	0.33	0.24	0.53
Rate:Camera8	0.18	0.00	0.00	0.00	0.00	0.25	0.40	0.00	0.27	0.19

**p-values**	Distress	Dog_force	Dog_move	Dog_pressure	Hum_Dog	Hum_Move	Movement	Obj_Int	Position	Space
(Intercept)	<0.01	<0.01	<0.01	<0.01	<0.01	<0.01	<0.01	0.00	0.00	<0.01
Rate	<0.01	<0.01	<0.01	0.00	<0.01	<0.01	<0.01	<0.01	<0.01	<0.01
Density1	<0.01	<0.01	0.00	<0.01	<0.01	<0.01	<0.01	0.00	0.00	<0.01
Density3	<0.01	<0.01	<0.01	<0.01	<0.01	<0.01	<0.01	<0.01	<0.01	<0.01
Density4	<0.01	0.29	0.03	0.00	<0.01	<0.01	<0.01	<0.01	0.00	<0.01
Density5	<0.01	<0.01	0.00	<0.01	<0.01	<0.01	<0.01	0.09	0.00	<0.01
Density6	<0.01	<0.01	<0.01	<0.01	0.21	<0.01	<0.01	0.00	0.54	<0.01
Num_start	0.30	0.81	0.24	0.28	0.11	0.04	0.00	0.01	0.67	0.00
Beh_cat.1Distress	0.00	0.00	0.00	0.00	0.01	0.00	0.44	0.02	0.00	0.00

Beh_cat.1Dog_force	0.00	0.00	0.00	0.00	0.00	0.03	0.08	0.46	0.00	0.00
Beh_cat.1Dog_move	0.00	0.00	0.00	0.00	0.00	0.01	0.20	0.02	0.00	0.00
Beh_cat.1Dog_pressure	0.00	0.00	0.00	0.00	0.00	0.03	0.25	0.29	0.04	0.01
Beh_cat.1Hum_Dog	0.17	0.01	0.00	0.00	0.00	0.01	0.61	0.43	0.01	0.24
Beh_cat.1Hum_Move	0.09	0.00	0.00	0.00	0.00	0.01	0.55	0.55	0.03	0.58
Beh_cat.1Movement	0.00	0.00	0.00	0.00	0.01	0.01	0.62	0.01	0.07	0.01
Beh_cat.1Obj_Int	0.02	0.00	0.00	0.00	0.00	0.01	0.04	0.04	0.00	0.05
Beh_cat.1Position	0.00	0.00	0.00	0.00	0.00	0.00	0.81	0.10	0.00	0.02
Beh_cat.1Space	0.00	0.01	0.00	0.01	0.00	0.05	0.83	0.03	0.05	0.00
Camera2	<0.01	0.00	0.00	<0.01	<0.01	0.48	<0.01	0.00	0.00	0.00
Camera3	<0.01	<0.01	0.00	0.00	0.00	<0.01	<0.01	<0.01	<0.01	<0.01
Camera4	<0.01	<0.01	0.00	<0.01	0.00	0.56	<0.01	0.00	0.31	0.14
Camera5	0.00	0.00	<0.01	0.00	0.11	<0.01	<0.01	0.00	<0.01	<0.01
Camera6	<0.01	<0.01	<0.01	<0.01	<0.01	0.00	<0.01	0.42	0.00	<0.01
Camera7	<0.01	<0.01	<0.01	0.00	<0.01	<0.01	<0.01	<0.01	<0.01	0.00
Camera8	<0.01	<0.01	<0.01	<0.01	<0.01	<0.01	0.02	<0.01	<0.01	<0.01
Stat_moveS	0.00	0.07	0.30	0.57	0.01	0.00	<0.01	0.01	0.01	0.00
Rate:Density1	<0.01	<0.01	<0.01	0.00	<0.01	<0.01	<0.01	<0.01	<0.01	<0.01
Rate:Density3	<0.01	<0.01	0.93	<0.01	<0.01	<0.01	<0.01	<0.01	0.00	<0.01
Rate:Density4	<0.01	0.00	0.00	0.00	<0.01	<0.01	<0.01	0.00	0.00	<0.01
Rate:Density5	<0.01	<0.01	<0.01	0.00	<0.01	<0.01	<0.01	<0.01	0.00	<0.01
Rate:Density6	<0.01	<0.01	<0.01	0.00	0.00	<0.01	<0.01	<0.01	<0.01	<0.01
Density1:Camera2	<0.01	0.00	0.55	<0.01	<0.01	0.00	<0.01	0.00	0.24	0.00
Density3:Camera2	<0.01	<0.01	NaN	<0.01	<0.01	<0.01	0.00	<0.01	<0.01	<0.01
Density4:Camera2	<0.01	<0.01	<0.01	0.14	NaN	<0.01	<0.01	<0.01	<0.01	<0.01
Density5:Camera2	<0.01	0.00	0.95	<0.01	<0.01	0.00	<0.01	0.05	0.39	0.00
Density6:Camera2	<0.01	0.00	<0.01	<0.01	<0.01	0.00	<0.01	0.00	0.17	0.00

Density1:Camera3	<0.01	0.03	0.00	<0.01	0.00	<0.01	<0.01	<0.01	<0.01	<0.01
Density3:Camera3	<0.01	<0.01	<0.01	<0.01	<0.01	<0.01	<0.01	<0.01	<0.01	<0.01
Density4:Camera3	<0.01	<0.01	<0.01	<0.01	<0.01	<0.01	<0.01	<0.01	<0.01	0.00
Density5:Camera3	<0.01	0.02	0.10	<0.01	<0.01	<0.01	<0.01	<0.01	<0.01	<0.01
Density6:Camera3	<0.01	0.00	<0.01	<0.01	<0.01	<0.01	<0.01	<0.01	<0.01	<0.01
Density1:Camera4	<0.01	<0.01	<0.01	<0.01	0.00	0.00	<0.01	<0.01	0.00	0.13
Density3:Camera4	0.00	<0.01	<0.01	<0.01	<0.01	<0.01	<0.01	<0.01	0.01	0.00
Density4:Camera4	0.00	<0.01	<0.01	<0.01	<0.01	0.20	<0.01	0.00	0.79	0.16
Density5:Camera4	0.00	<0.01	<0.01	<0.01	<0.01	0.00	<0.01	0.00	0.03	0.13
Density6:Camera4	<0.01	<0.01	<0.01	0.00	<0.01	0.19	<0.01	0.00	0.21	0.01
Density1:Camera5	0.03	0.00	<0.01	0.00	0.00	<0.01	<0.01	0.00	0.00	<0.01
Density3:Camera5	<0.01	NaN	<0.01	<0.01	<0.01	<0.01	<0.01	<0.01	<0.01	<0.01
Density4:Camera5	<0.01	<0.01	<0.01	<0.01	<0.01	<0.01	<0.01	<0.01	<0.01	<0.01
Density5:Camera5	0.31	0.00	0.00	0.30	<0.01	0.16	<0.01	0.58	0.38	<0.01
Density6:Camera5	0.00	<0.01	<0.01	<0.01	<0.01	0.00	<0.01	0.00	0.00	<0.01
Density1:Camera6	<0.01	<0.01	<0.01	<0.01	<0.01	0.00	<0.01	0.27	<0.01	<0.01
Density3:Camera6	<0.01	NaN	<0.01	<0.01	NaN	NaN	<0.01	NaN	<0.01	<0.01
Density4:Camera6	<0.01	<0.01	<0.01	<0.01	NaN	<0.01	<0.01	<0.01	<0.01	<0.01
Density5:Camera6	0.00	0.00	<0.01	0.00	<0.01	0.00	<0.01	0.00	<0.01	<0.01
Density6:Camera6	<0.01	<0.01	<0.01	<0.01	<0.01	0.05	<0.01	0.01	0.00	<0.01
Density1:Camera7	<0.01	0.00	0.00	<0.01	<0.01	<0.01	<0.01	0.13	<0.01	0.00
Density3:Camera7	0.00	<0.01	<0.01	<0.01	<0.01	<0.01	<0.01	<0.01	<0.01	<0.01
Density4:Camera7	<0.01	<0.01	0.00	0.00	<0.01	<0.01	<0.01	<0.01	<0.01	0.00
Density5:Camera7	<0.01	0.00	0.00	<0.01	<0.01	<0.01	<0.01	0.00	<0.01	0.92
Density6:Camera7	<0.01	0.00	<0.01	<0.01	<0.01	<0.01	<0.01	0.24	0.00	0.00
Density1:Camera8	<0.01	<0.01	<0.01	<0.01	<0.01	<0.01	<0.01	<0.01	<0.01	<0.01
Density3:Camera8	0.76	NaN	NaN	<0.01	<0.01	<0.01	<0.01	<0.01	0.00	<0.01

Density4:Camera8	<0.01	<0.01	<0.01	<0.01	<0.01	<0.01	<0.01	<0.01	<0.01	<0.01
Density5:Camera8	<0.01	<0.01	<0.01	<0.01	<0.01	<0.01	<0.01	<0.01	<0.01	<0.01
Density6:Camera8	<0.01	<0.01	<0.01	<0.01	<0.01	<0.01	<0.01	<0.01	0.00	<0.01
Rate:Camera2	0.05	0.03	0.00	0.00	<0.01	0.00	0.36	0.00	0.00	0.06
Rate:Camera3	<0.01	0.00	0.04	0.52	<0.01	<0.01	<0.01	0.00	<0.01	0.00
Rate:Camera4	0.02	0.34	0.93	0.61	<0.01	0.58	0.19	0.36	0.23	0.47
Rate:Camera5	0.33	0.00	0.00	0.00	<0.01	0.66	0.04	0.21	0.30	0.28
Rate:Camera6	0.00	0.38	0.53	0.24	<0.01	0.05	0.38	0.01	0.11	0.01
Rate:Camera7	0.00	0.00	0.00	0.00	<0.01	0.00	0.62	0.81	0.00	0.00
Rate:Camera8	<0.01	<0.01	<0.01	<0.01	<0.01	<0.01	<0.01	<0.01	<0.01	<0.01

Factor	Estimate	Std. Error	t-value
(Intercept)	**3.83**	**0.52**	**7.43**
Distress	0.02	0.02	1.15
Dog_force	**-0.11**	**0.03**	**-4.32**
Dog_movement	-0.04	0.02	-1.77
Dog_pressure	**-0.11**	**0.02**	**-4.75**
Human-dog int.	0.05	0.04	1.25
Human_movement	0.02	0.02	1.25
Sheep_movement	**0.13**	**0.04**	**3.44**
Object_int.	0.06	0.03	1.71
Position	0.01	0.02	0.38
Space	**0.14**	**0.02**	**5.89**
No sheep (Density1)	0.04	0.04	1.04
Loose (Density3)	0.07	0.08	0.87
Moderate (Density4)	**0.17**	**0.05**	**3.87**
Bunched (Density5)	**0.10**	**0.04**	**2.17**
Packed (Density6)	**0.22**	**0.05**	**4.72**
Number_start	**-<0.01**	**<0.01**	**-2.61**
Density1: Num_start	**0.01**	**<0.01**	**4.19**
Density3: Num_start	**0.01**	**<0.01**	**2.63**
Density4: Num_start	<0.01	<0.01	1.08
Density5: Num_start	**<0.01**	**<0.01**	**3.11**
Density6: Num_start	**<0.01**	**<0.01**	**3.79**
